# Expression patterns of long non-coding RNAs in peripheral blood mononuclear cells of non-segmental vitiligo

**DOI:** 10.1097/MD.0000000000028399

**Published:** 2021-12-23

**Authors:** Shulan Zhang, Xinyue Yang, Zhibin Zhang, Yifeng Xiong, Yingpeng Zhang, Chunming Li, Ougen Liu, Xiaoyan Wang, Yating Peng

**Affiliations:** a Department of Dermatology, The Second Affiliated Hospital of Nanchang University, Nanchang, Jiangxi, China; b Department of Dermatology, The First Affiliated Hospital of Nanchang University, Nanchang, Jiangxi, China; c Department of Pathology, The First Affiliated Hospital of Nanchang University, Nanchang, Jiangxi, China.

**Keywords:** bioinformatics, lncRNAs, non-segmental vitiligo, peripheral blood mononuclear cells, RNA sequencing

## Abstract

**Objective:**

We explored the patterns of long non-coding RNA (lncRNA) expression in peripheral blood mononuclear cells (PBMCs) from patients with non-segmental vitiligo.

**Methods:**

We used high-throughput RNA sequencing technology to generate sequence data from five patients with non-segmental vitiligo alongside five normal healthy individuals, and then performed bioinformatics analyses to detect the differential expression of lncRNA in PBMCs. Gene Ontology (GO) and pathway analyses were performed for functional annotation, and quantitative real-time polymerase chain reaction (qRT-PCR) was used to verify gene expression. Target miRNAs and mRNAs of differentially expressed lncRNAs were predicted using bioinformatics analysis.

**Results:**

A total of 292 lncRNAs were differentially expressed in non-segmental vitiligo (fold change ≥ 2.0, *P* < .05), of which 171 were upregulated and 121 were downregulated. Six differentially expressed lncRNAs were selected, namely ENST00000460164.1, ENST00000393264.2, NR_-_046211.1, NR_-_135491.1, NR_-_135320.1, and ENST00000381108.3, for validation by qRT-PCR. The results showed that ENST00000460164.1 and NR_-_046211.1 were highly expressed in PBMCs of non-segmental vitiligo. An lncRNA-miRNA-mRNA network containing two lncRNAs, 17 miRNAs, and 223 mRNAs was constructed.

**Conclusion:**

Our results revealed patterns of differentially expressed lncRNAs in the PBMCs of non-segmental vitiligo individuals. ENST00000460164.1, and NR_-_046211.1 may be potential biomarkers and drug targets for the treatment of non-segmental vitiligo.

## 1 Introduction

Vitiligo, a skin immune disease mediated by T cells,^[1]^ has an incidence of about 0.5% to 2%, mostly in young people.^[2]^ Peripheral blood mononuclear cells (PBMCs) consist of leukocytes in the blood circulation that have a single round nucleus such as lymphocytes (T cells, B cells), natural killer cells, monocytes and dendritic cells, and constitute a critical component of the immune system.

The skin is a large and important peripheral lymphoid organ in which many immune cells migrate from the blood circulation. In patients with vitiligo, PBMCs can secrete proinflammatory cytokines such as IL-1β, IL-6, IL-8, and tumor necrosis factor (TNF)-a,^[3]^ and infiltrate around the lesions of vitiligo,^[4]^ suggesting that PBMCs play important roles in the pathogenesis of vitiligo.

Noncoding RNAs, such as long non-coding RNAs (lncRNAs), microRNAs (miRNAs), and circular RNAs (circRNAs), play an important role in the regulation of gene expression. Long non-coding RNAs (lncRNAs), a type of non-coding RNA with a length >200 bp, are ubiquitous in mammals. Sometimes, the stability of lncRNA is reduced by the interaction of targeted microRNA.^[5]^ LncRNA can also be used as a sponge or molecular bait for microRNA.^[6]^ In addition, lncRNA can compete with microRNA for target mRNA.^[7]^ Moreover, some lncRNAs can produce miRNAs to silence the target messenger RNA (mRNA).^[8]^

Numerous studies have shown that non-coding RNAs play a regulatory role in the pathogenesis of vitiligo.^[9]^ miRNA differential expression profiles of miRNAs were obtained from skin lesions, melanocytes, peripheral blood mononuclear cells (PBMCs), and serum samples from patients with vitiligo.^[10–12]^ miRNA microarray analysis was used to compare the miRNA expression profiles of PBMCs in the peripheral blood of patients with NSV and those of normal controls.^[11,13]^

To the best of our knowledge, there are no studies on the patterns of lncRNA expression in PBMC of vitiligo, and the pathogenesis of vitiligo remains unknown. We hypothesized that lncRNAs in PBMCs may be involved in the pathogenesis of vitiligo. In this study, we used high-throughput RNA sequencing to reveal differentially expressed lncRNAs in PBMCs from patients with advanced non-segmental vitiligo and healthy individuals. We then performed qRT-PCR to validate the expression levels of the selected differentially expressed lncRNAs.

## 2 Materials and methods

### 2.1 Sample collection

Thirty patients with advanced non-segmental vitiligo and 30 healthy individuals were enrolled from the outpatient department of the Second Affiliated Hospital of Nanchang University. Among them, 5 patients with vitiligo and 5 healthy controls were performed high-throughput sequencing and qRT-PCR verification. All patients or their family members provided informed consent prior to inclusion in the study, which was approved by the medical ethics committee of the Second Affiliated Hospital of Nanchang University, according to the Declaration of Helsinki.

According to the manufacturer's instructions (Amersham Pharmacia, Uppsala, Sweden), PBMCs were purified using standard Ficoll-Paque gradient centrifugation. Briefly, 4 mL Ficoll-Paque gradient pipette was transferred into two 15 mL centrifuge tubes. The heparinized blood was diluted 1:1 in phosphate buffered saline (PBS) and carefully stratified on a Ficoll-Paque gradient (9–10 mL/tube). The tubes were centrifuged for 20 min at 1020×*g*. The cell interface layer was harvested carefully, and the cells were washed twice in PBS (for 10 min at 640×*g* followed by 10 min at 470×*g*), then were added 20 times of Trizol and was blown until transparent, which could be frozen at −80°C before sample delivery.

The diagnostic criteria were in line with the 2013 European guidelines for the management of vitiligo.^[14]^ The clinical data of the enrolled patients are summarized in Table 1. Patients aged 18 to 60 years old and with a course of 3 to 6 months who met the diagnostic criteria were included in this study. Patients with the following conditions were excluded.

1. Patients systemic use of glucocorticoids, immunosuppressive drugs, various topical drugs, and phototherapy 4 weeks before treatment.
2. Patients suffer from heart, liver, spleen, lung, kidney, other systemic diseases, mental diseases, other autoimmune diseases, and skin malignant tumors.

**Table 1:** Clinical characteristics of vitiligo patients and healthy controls.

Characteristics	Vitiligo	Normal people
Total number	30	30
Gender (female/male)	12/18	12/18
Age (y), mean ± SD	45.70 ± 10.16	47.7 ± 11.1
LncRNA microarray	5	5
Age (y), mean ± SD	25.40 ± 5.68	26.20 ± 3.97
qRT-PCR	30	30

Healthy individuals aged 18 to 60 years old without vitiligo, systemic diseases, mental illness, other autoimmune diseases, and skin malignancies were included in the control group.

### 2.2 High-throughput sequencing

Peripheral blood samples from five vitiligo patients and five healthy controls were subjected to mononuclear cell extraction within 2 hours, followed by high-throughput lncRNA sequencing. Briefly, total RNA was extracted from the samples, rRNA was removed using a ribosome removal kit (Illumina, USA), and then fragmented (to an average fragment length of ∼200 nt). Reverse transcription was performed to synthesize single-stranded cDNA, followed by the synthesis and purification of double-stranded cDNA. The ends were repaired and combined using add adapter primers, subjected to PCR amplification (Takara Bio Group, Japan) and purification, quality inspection of the libraries, and sequencing.

### 2.3 lncRNA sequencing analysis

Total RNA from peripheral blood mononuclear cells of five matched patients with advanced vitiligo and five healthy individuals was extracted and purified using the Trizol kit (Life Technologies, USA). Preliminary evaluation of RNA degradation and contamination with genomic DNA was performed using agarose gel electrophoresis using a Nanodrop (Thermo Fisher Company, USA) to ascertain purity of the total RNA, and to evaluate whether there was protein or phenol contamination based on the OD260/280 ratio, and salt ion or carbohydrate contamination based on the OD260/230 ratio, and the Agilent 2200 Bioanalyzer (Agilent Technologies, USA) was used to detect the integrity of the RNA and calculate the RNA integrity index (RIN value) based on the RNA peak graph, requiring a RIN value > 7.0. Approximately 3 μg of the total RNA from each sample was subjected to ribosomal RNA using the Epicenter Ribo-Zero rRNA Removal Kit (Illumina, USA), the purified RNA treated with RNase R (Epicenter, USA), and purified with Trizol. RNA-seq libraries were prepared using the NEBNext Ultra RNA Library Prep Kit (NEB, USA), which were subsequently sequenced on the Illumina HiSeq 3000 platform at Guangzhou Ruibo Co Ltd (Illumina, USA). RNAseq raw data have been submitted to public database GEO, the GEO number is GSE186928.

### 2.4 Analysis of lncRNA sequence data

Raw sequence reads were filtered to remove adapters, low-quality reads, and ribosomal RNA by HISAT2 software. Clean reads were mapped to the reference genome to obtain a BAM file by improved BWT algorithm. GO and Kyoto encyclopedia of genes and genomes (KEGG) analysis were performed to further annotated the functions and signal transduction pathways of these genes (|log2FoldChange|>2 and *P* < .05), with *P* < .05 as the threshold to reveal biological functions and pathways.

### 2.5 Prediction of lncRNA-miRNA interaction

TargetScan, MiRDB, and mirTarbase software were used to predict the miRNA binding sites on lncRNAs. Points of intersection were analyzed, and the joint predictions from the three softwares were used as the final prediction results. These results were used to construct the lncRNA-miRNA networks.

### 2.6 qRT-PCR

Peripheral blood mononuclear cells from 30 patients with vitiligo and 30 healthy controls were extracted, total RNA extraction and cDNA synthesis were performed as previously described.^[15]^ Then qRT-PCR amplification was performed using the 7900 HT Sequence Detection System (ABI, Foster City, CA), using the SYBR Green kit (Takara Bio Group, Japan) and specific primers targeting candidate lncRNAs. The relative expression was confirmed using the 2-ΔΔCt method. β-Actin was used as an internal amplification control.qPCR primer sequences are listed in Table 2.

**Table 2:** Primers used for qRT-PCR analysis of lncRNAs.

LncRNA	Forward primer	Reverse primer
ENST00000460164	F:5’ CAAAACAGGAAGGGCTGAGAC 3’	R:5’ GAAGTAGTCCTTGACCAGGCAG 3’
ENST00000393264	F:5’ TTTGCCTCTGAAGGTGCTCA 3’	R:5’ TTGCACGGTACAGTTTGCAG 3’
NR_046211	F:5’ CGGCTGTCCTACAGTCCTCA 3’	R:5’ GCTTGTGATCTACGTTGCAGGT 3’
ENST00000381108.3	F:5’ CCAGAGGCAAAGGTATCGTCC 3’	R:5’ GAGTAAGTTGAGGGTGTGGGAA 3’
NR_135491.1	F:5’ TGTGGCACCACAGGATATTCTA 3’	R:5’ TGTGGCACCACAGGATATTCTA 3’
NR_135320.1	F:5’ GCATTTGGGAAGTAAAACACTATCC 3’	R:5’ GCATTTGGGAAGTAAAACACTATCC 3’

### 2.7 Prediction of lncRNA-miRNA-mRNA interaction

We used Cytoscape 3.6.1 to construct a lncRNA-miRNA-mRNA network based on the ceRNA hypothesis and determined their interaction. Briefly, we chose a known target miRNA for each lncRNA, then applied MiRWalk v3.0 (http://mirwalk.umm.uni-heidelberg.de/) and strict screening conditions, including three prediction algorithms (Targetscan, MiRDB, and mirTarbase) to predict downstream target genes. Finally, we described the ceRNA network using two clearly differentially expressed lncRNAs and predicted miRNAs and mRNAs that had been verified.

## 3 Statistical analysis

All data were analyzed using SPSS software (version 25.0; SPSS, Chicago, IL) and expressed as mean ± standard deviation (M ± SD). Comparisons between the groups were performed using Student's *t* test. Data were considered statistically significant at *P* < .05.

## 4 Results

### 4.1 lncRNA expression profile

**Figure 1:**
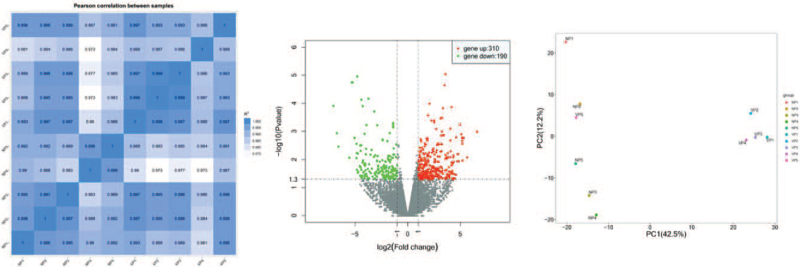
Expression patterns of lncRNAs in progressive non-segmental VP and NP based on RNA-seq data. (A) A scatter plot showing levels of lncRNAs in all samples. Darker color denotes higher correlation coefficients (blue squares, pale blue squares, and white squares). (B) Volcano plot showing differentially expressed lncRNAs between groups. Red and green dots represent up-regulated and downregulated lncRNAs, respectively. (C) PCA plot showing the similarity of samples. PCA is a mathematical algorithm, which can reduce the data dimension and retain the vast majority of variables in the data set. NP = normal people, PCA = Principal component analysis, VP = vitiligo people.

**Figure 2:**
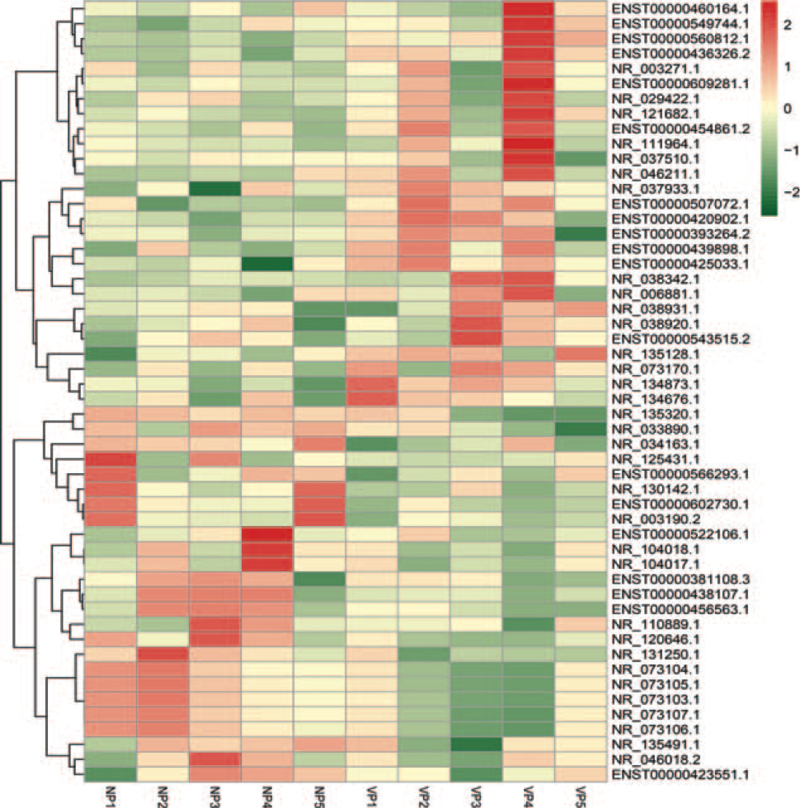
Profiles of lncRNA expression in progressive non-segmental VP and NP. Hierarchical grouping based on a heat map. Red and green colors represent up-regulated and downregulated lncRNAs, respectively. NP = normal people, VP = vitiligo people.

**Figure 3:**
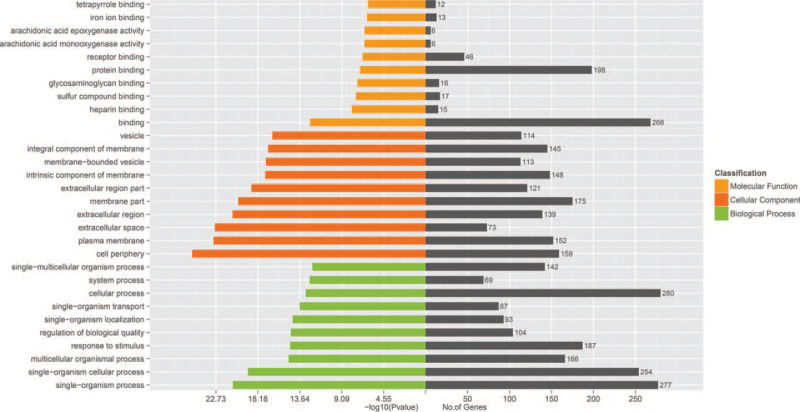
The top 30 GO enrichment terms. GO assessment was conducted on the parental genes of lncRNAs with different expression levels (|log2Fold_Change| ≥ 2, *P* < .05). Statistically significant biological functions and pathways were obtained using a threshold of *P* < .05.

**Figure 4:**
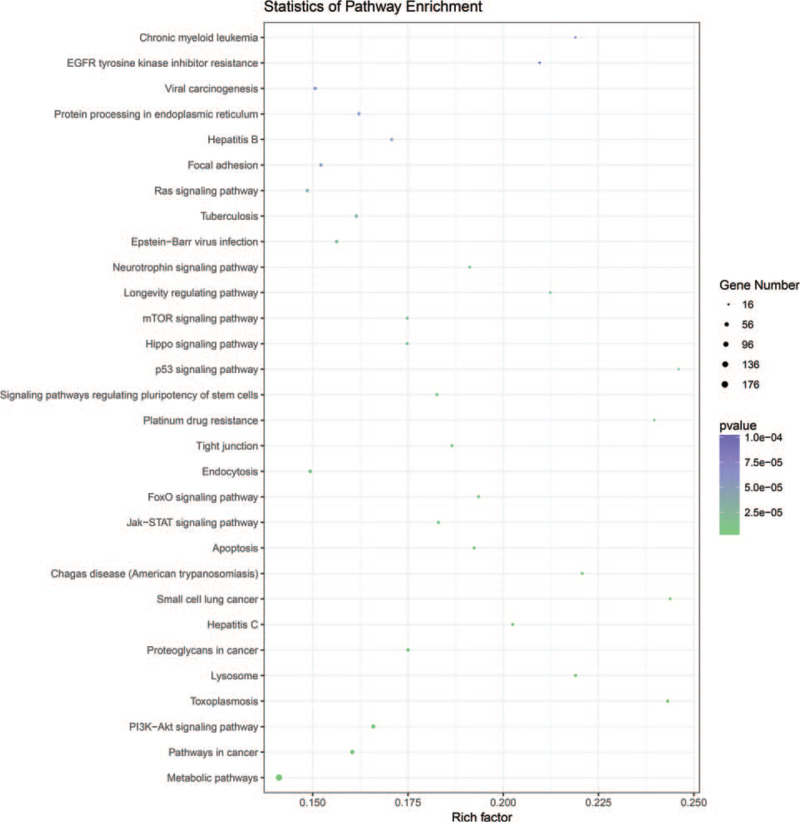
The top 30 KEGG pathways are enriched in terms. The size of the dots in the graph indicates the number of enriched differentially expressed genes, and the color indicates the *P* value.

Distinguishable lncRNA expression patterns between the progressive non-segmental vitiligo people (VP) group and normal people (NP) group are shown using a scatter plot (Fig. 1A). The volcano graph was used to select differentially expressed lncRNAs between VP group and NP group (Fig. 1B). The red and green points represented upregulated and downregulated lncRNAs in the two groups. The black points were behalf of no statistically significant difference lncRNAs between the 2 groups. Afterward, we identified 500 differentially expressed lncRNAs (310 upregulated and 190 downregulated lncRNAs, fold change ≥ 1.3, *P* < .05) in the VP group compared with the NP group. And a total of 292 differentially expressed lncRNAs were detected (fold change ≥ 2.0, *P* < .05) in PBMCs from patients with non-segmental vitiligo and healthy individuals. Of these, 171 were upregulated and 121 were downregulated. The similarity of samples are shown using a principal component analysis (PCA) plot (Fig. 1C). Hierarchical clustering was used to identify differentially expressed lncRNAs between the two groups (Fig. 2). GO analysis revealed genes focused on biological processes, molecular functions, and cellular components. Specifically, most of the target genes were enriched in binding, intracellular, and cellular processes (Fig. 3). KEGG pathway analysis revealed genes mainly involved in a series of biological processes, such as metabolism, apoptosis, Jak-STAT, and the PI3K-Akt signaling pathway (Fig. 4).

### 4.2 Construction of lncRNA/miRNA interaction network

**Figure 5:**
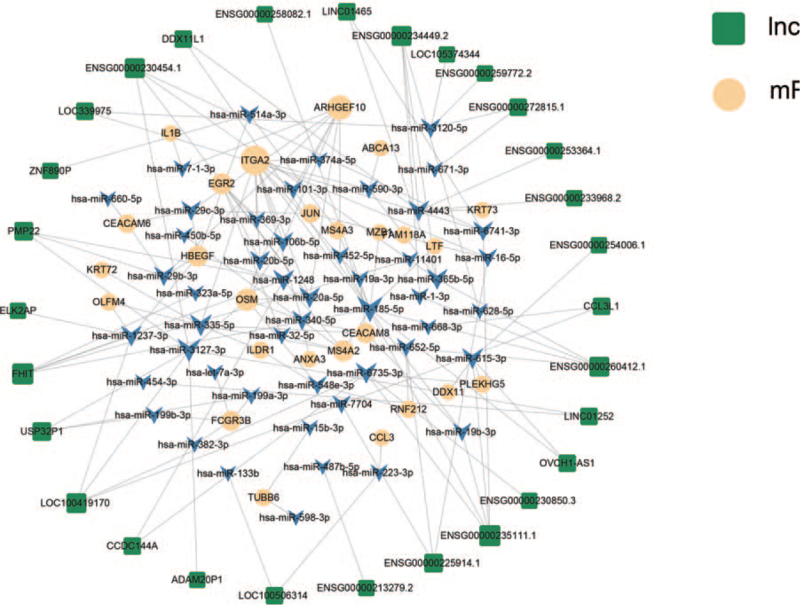
A network of the top 29 lncRNAs constructed using Cytoscape3.6.1. Green squares denote the lncRNAs, blue arrows indicate their target miRNAs (only top 49 included), whereas yellow circles denote their target mRNAs (only top 26 included).

In order to find the downstream miRNAs that can bind to lncRNAs, Targetscan, MiRDB, and mirTarbase software were used to predict miRNA binding sites on the lncRNAs and reveal lncRNA function. The results showed an intersection of lncRNAs in a network that included 29 lncRNAs, 49 miRNAs, and 26 mRNAs. The network showed that some lncRNAs were predicted to bind to several target miRNAs and some miRNAs were predicted to bind to several target genes. Moreover, one miRNA may bind to multiple different lncRNAs (Fig. 5). The differentially expressed lncRNAs and their predicted miRNAs were graphically presented using Cytoscape 3.6.1 software.

### 4.3 Validation of gene expression

**Figure 6:**
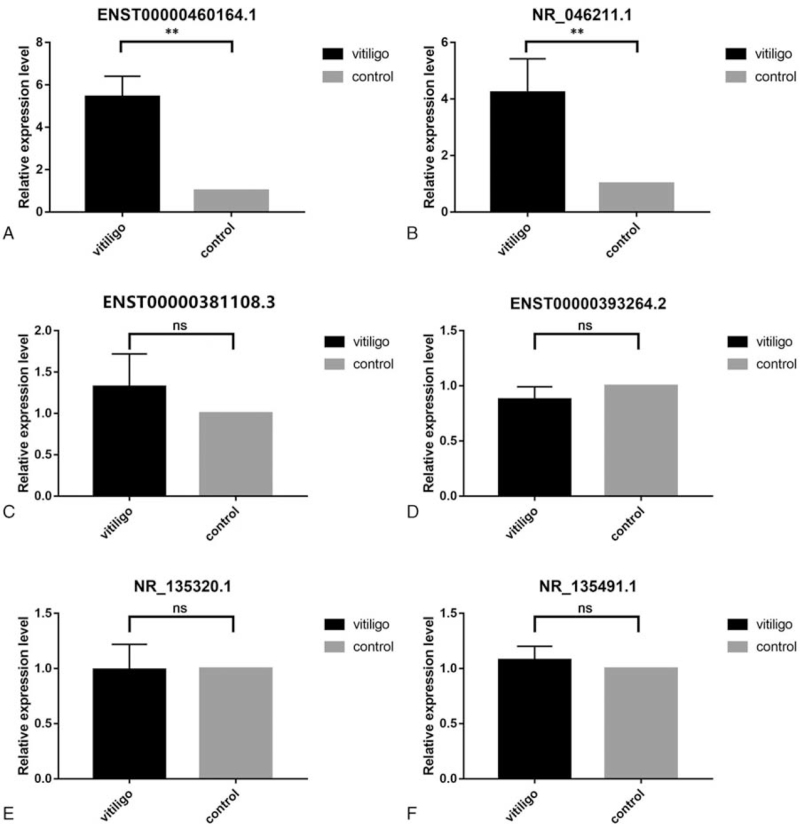
The expression level of lncRNAs. The expression of ENST00000460164.1 (A) and NR_-_046211.1 (B) in 30 vitiligo patients following qRT-PCR (^∗∗^*P* < .01). The expression of ENST00000381108.3 (C), ENST00000393264.2 (D), NR_-_135320.1 (E), and NR_-_135491.1 (F) in 30 vitiligo patients following qRT-PCR (ns: *P* > .05).

Six highly differentially expressed lncRNAs (ENST00000460164.1, ENST00000393264.2, NR_-_046211.1, NR_-_135491.1, NR_-_135320.1, and ENST00000381108.3) were selected for qRT-PCR validation in the non-segmental vitiligo and control groups. The results showed that ENST00000460164.1 and NR_-_046211.1 were highly expressed in PBMCs of non-segmental vitiligo compare to the healthy controls (*P* < .01), and ENST00000393264.2, NR_-_135491.1, NR_-_135320.1, ENST00000381108.3 were no significant difference between non-segmental vitiligo and control groups (*P* > .05) (Fig. 6), which is consistent with the RNA-sequence data.

### 4.4 lncRNA-miRNA-mRNA network prediction

**Figure 7:**
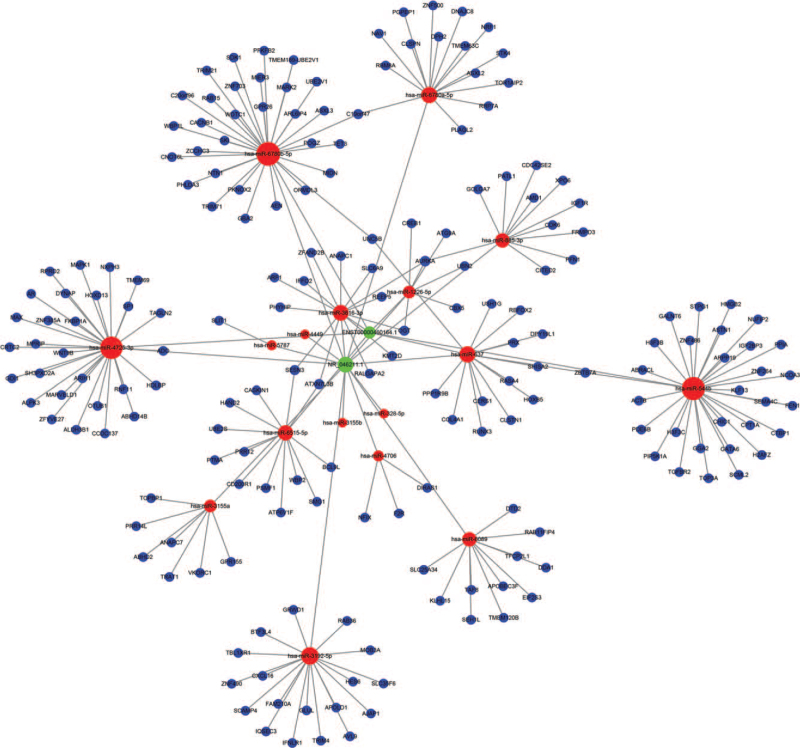
The lncRNA-miRNA-mRNA network containing 17 miRNAs and 223 mRNAs. The 17 predicted target miRNAs (red circles) and mRNAs (blue circles) of differentially expressed lncRNAs (green squares) were identified using Cytoscape3.6.1.

In order to predict the downstream genes of these two lncRNAs, we constructed a ceRNA-based network of the two distinctly differentially expressed lncRNAs, ENST00000460164.1 and NR_-_046211.1, in PBMCs of non-segmental advanced vitiligo. This network contained two lncRNAs, 17 miRNAs that may bind to these lncRNAs, and 223 downstream target mRNAs of the miRNAs (Fig. 7).

## 5 Discussion

According to the Consensus on the treatment of vitiligo—Brazilian Society of Dermatology, vitiligo can be categorized into clinical classes, including segmental vitiligo and non-segmental vitiligo.^[16]^ However, non-segmental vitiligo is the most common type. NSV and SV were believed to have distinct underlying pathogenetic mechanisms due to their different clinical presentations, with the neuronal hypothesis or somatic mosaicism favored for the segmental form.^[17]^ NSV shows unstable disease course, while SV is often stable a few months after onset. Therefore, we chose NSV for further research.

The results of the present study revealed several differentially expressed lncRNAs (171 upregulated and 121 downregulated) in PBMCs from non-segmental advanced vitiligo and normal individuals. GO and KEGG analyses revealed that most of the identified genes and pathways in non-segmental vitiligo cases were associated with metabolism, apoptosis, Jak-STAT, and PI3K-Akt signaling pathways. This suggests that lncRNAs associated with these biological processes may be involved in the pathogenesis of non-segmental vitiligo. qRT-PCR verified that two lncRNAs (ENST00000460164.1 and NR_-_046211.1) were significantly upregulated in non-segmental vitiligo specimens, which was consistent with the RNA sequencing data. These two lncRNAs may play crucial roles in the occurrence and development of non-segmental vitiligo.

Previous studies have demonstrated that lncRNAs are involved in the occurrence and development of many diseases and can be used as biomarkers for the early diagnosis and prognosis of diseases. Moreover, recent studies have suggested that lncRNAs may be candidates for targeted therapy aimed at alleviating or even curing diseases,^[18,19]^ while other studies have reported the key role lncRNAs play in regulating miRNA expression in many diseases.^[20,21]^ In fact, they act as a scaffold that coordinates specific regulatory functions^[22]^ or compete for endogenous RNA.^[23]^ In Hirschsprung's disease, lncRNAFAL1 was found to regulate AKT1 expression by competing with miR-637 as a ceRNA.^[24]^

In addition, studies found that lncRNAs may play a regulatory role in vitiligo. It has been reported that LncRNA TUG1 was significantly downregulated in serum of vitiligo patients.^[25]^ Other studies suggested that lncRNA MALAT1 was upregulated in the epidermis lesion of vitiligo and may protection from UV mediated DNA damage by function as a miR-211 suppressor.^[26]^ However, our high throughput sequencing data showed that there was no significant change in the expression of LncRNA TUG1 and lncRNA MALAT1 in PBMC of vitiligo patients. The possible reason may be that these lncRNAs come from different types of samples. The samples to be detected in this study were PBMC, while the other two studies were serum and epidermis respectively.

In the present study, high-throughput RNA sequencing and qRT-PCR results revealed significantly upregulation of two lncRNAs (ENST0000460164.1 and NR_-_046211.1) in non-segmental vitiligo. miR-328 and miR-637 were predicted to be the most significant binding sites for these two lncRNAs. Among them, ENST0000460164.1 was predicted to bind with mir-637 via a bioinformatics method. Bioinformatics analysis showed that mir-637 was predicted to bind to IL16 and IL34. Therefore, ENST0000460164.1 may act through the ENST0000460164.1/mir-637/IL16 or ENST0000460164.1/mir-637/IL34 axis in vitiligo. NR_-_046211.1 was predicted to bind with mir-637 and mir-328 via a bioinformatics method. miR-328 was downregulated in skin lesions and was found to regulate the oxidative stress mechanism in vitiligo patients by targeting IL1β.^[9]^ Researches showed that the levels of IL1β was significantly upregulated in vitiligo patients.^[27–29]^ In addition, bioinformatics analysis showed that mir-328 was predicted to bind to IL1β. Therefore, NR_-_046211.1 may regulate the pathogenesis of vitiligo through NR_-_046211.1/mir-328/IL1β axis, but NR_-_046211.1/mir-637/IL16, and NR_-_046211.1/mir-637/IL34 axis need further confirmation.

These results provide new insights into the underlying mechanisms of non-segmental vitiligo. However, this is still a preliminary study and needs to be explored from the following aspects. In our future study, we will trying to analyze the expression of lncRNAs in the inflammatory cells of the rash. In addition, future studies are required to screen for more differentially expressed lncRNAs in larger sample sizes. Further validation of the role of ENST00000460164.1 and NR_-_046211.1 is required, mainly through knockdown or overexpression experiments, while detailed elucidation of the underlying mechanism of lncRNA-miRNA-mRNA action in advanced non-segmental vitiligo is imperative.

## Acknowledgments

We are very grateful to all participants in the study.

## Author contributions

**Conceptualization:** Xinyue Yang, Ougen Liu.

**Data curation:** Zhibin Zhang.

**Formal analysis:** Yifeng Xiong.

**Methodology:** Yingpeng Zhang.

**Project administration:** Chunming Li.

**Resources:** Xiaoyan Wang.

**Writing – original draft:** Shulan Zhang.

**Writing – review & editing:** yating Peng.
